# The Crohn’s Disease Exclusion Diet: A Comprehensive Review of Evidence, Implementation Strategies, Practical Guidance, and Future Directions

**DOI:** 10.1093/ibd/izad255

**Published:** 2023-11-18

**Authors:** Rotem Sigall Boneh, Catherine Westoby, Ilan Oseran, Chen Sarbagili-Shabat, Lindsey G Albenberg, Paolo Lionetti, Víctor Manuel Navas-López, Javier Martín-de-Carpi, Henit Yanai, Nitsan Maharshak, Johan Van Limbergen, Eytan Wine

**Affiliations:** Pediatric Gastroenterology and Nutrition Unit, The E. Wolfson Medical Center, Holon, Israel; Tytgat Institute for Liver and Intestinal Research, Amsterdam Gastroenterology Endocrinology and Metabolism, University of Amsterdam, Amsterdam, the Netherlands; Department of Nutrition and Dietetics, University Hospital Southampton NHS Foundation Trust, Southampton, UK; Department of Psychology, Ben-Gurion University of the Negev, Beer-Sheva, Israel; Pediatric Gastroenterology and Nutrition Unit, The E. Wolfson Medical Center, Holon, Israel; The Sackler Faculty of Medicine, Tel-Aviv University, Tel-Aviv, Israel; Division of Gastroenterology, Hepatology, and Nutrition, Children’s Hospital of Philadelphia, Philadelphia, PA, USA; Department Neurofarba, University of Florence, Meyer children’s Hospital IRCCS, Florence, Italy; Pediatric Gastroenterology and Nutrition Unit. Hospital Regional Universitario de Málaga. IBIMA. Málaga, Spain; Paediatric Gastroenterology, Hepatology and Nutrition Department, Hospital Sant Joan de Déu, Barcelona, Spain; IBD center, Division of Gastroenterology, Rabin Medical center, Petach Tikva, Israel; affiliated with the Faculty of Medicine, Tel Aviv University, Tel Aviv, Israel; Tel Aviv Medical Center, affiliated to the Faculty of Medicine, Tel Aviv University, Tel Aviv, Israel; Department of Paediatric Gastroenterology and Nutrition, Amsterdam University Medical Centers, Emma Children’s Hospital, Amsterdam, the Netherlands; Amsterdam Public Health research institute, University of Amsterdam, Amsterdam, the Netherlands; Division of Pediatric Gastroenterology, Department of Pediatrics, University of Alberta, Edmonton, AB, Canada

**Keywords:** nutritional therapy, Crohn’s disease, gut microbiome, exclusion diets

## Abstract

Dietary therapy is increasingly recognized for the management of Crohn’s disease (CD) over recent years, including the use of exclusive enteral nutrition (EEN) as first-line therapy for pediatric CD according to current guidelines. The Crohn’s disease exclusion diet (CDED) is a whole-food diet designed to reduce exposure to dietary components that are potentially pro-inflammatory, mediated by negative effects on the gut microbiota, immune response, and the intestinal barrier. The CDED has emerged as a valid alternative to EEN with cumulative evidence, including randomized controlled trials, supporting use for induction of remission and possibly maintenance in children and adults. We gathered a group of multidisciplinary experts, including pediatric and adult gastroenterologists, inflammatory bowel diseases (IBD) expert dietitians, and a psychologist to discuss the evidence, identify gaps, and provide insights into improving the use of CDED based on a comprehensive review of CDED literature and professional experience. This article reviews the management of CDED in both children and adults, long-term aspects of CDED, indications and contraindications, selecting the best candidates, identifying challenges with CDED, globalization, the role of the multidisciplinary team, especially of dietitian, and future directions. We concluded that CDED is an established dietary therapy that could serve as an alternative to EEN in many pediatric and adult cases, especially with mild to moderate disease. In severe disease, complicated phenotypes, or with extraintestinal involvement, CDED should be considered on a case-by-case basis, according to physician and dietitians’ discretion. More studies are warranted to assess the efficacy of CDED in different scenarios.

**Key Messages**

- **What is already known?** The Crohn’s disease exclusion diet (CDED) is an established dietary therapy for induction of remission in children and adults.
- **What is new here?** In this article, a group of experts comprehensively review evidence for CDED, bridging gaps in its understanding; we cover management across age groups, long-term aspects, candidate selection, challenges, and the multidisciplinary approach.
- **How can this study help patient care?** This review offers healthcare providers guidance on implementing the CDED, including its appropriate application, practical advice, challenges, and potential solutions; we anticipate that this knowledge will enhance nutritional care, improving patient outcomes.

## Introduction and Setting the Stage

Diet likely has a key role in the pathogenesis of inflammatory bowel diseases (IBD)^1^; however, very few diets have actually demonstrated clear therapeutic benefits.^2^ The best known and most evidence-supported dietary therapy for IBD is exclusive enteral nutrition (EEN),^3^ which is currently considered the first-line therapy for induction of remission in pediatric Crohn’s disease (CD), following the European Crohn’s and Colitis Organization (ECCO) and European Society for Paediatric Gastroenterology, Hepatology and Nutrition (ESPGHAN) guidelines.^4^ The Crohn’s disease exclusion diet (CDED) has emerged as a potential alternative, given EEN’s significant challenges and barriers.^5^ Several groups have recognized the potential of CDED to serve as an alternative to EEN in the management of luminal CD among children and adults,^6^ and the recent European Society of Parenteral and Enteral Nutrition (ESPEN) guidelines state that CDED might be considered as an alternative to EEN in both children and adults.^7^ Both diets, and other diets that have shown an impact in IBD, follow the principle of exclusion, guided by strong evidence that some foods can be harmful in IBD.^8,9^ Other mechanisms of action of CDED, related to the ability to modify the microbiota and their metabolic impacts, are reviewed here.^10-13^

Since the publication of the CDED randomized controlled trial (RCT) in 2019,^14^ additional studies have confirmed its effectiveness and tolerability^15-17^; however, many challenges still remain. The aim of this article is to review and discuss the evidence on CDED published over the last few years, to share experience and expert opinion, and to identify and reflect on some of the major remaining challenges and research gaps. This effort will help set the agenda for both research and clinical priorities and hopefully stimulate collaborations to expand the field of dietary therapy in IBD and improve patient outcomes.

## Methods

This project was initiated as a narrative review by the lead authors (R.S.B., E.W.) who suggested a list of relevant topics based on the currently available evidence and frequent questions raised by clinicians. A diverse array of experts was then invited to first identify and agree upon the relevant topics by consensus. Experts were assigned to specific working groups to review the published literature for each of the chosen topics without particular inclusion or exclusion criteria, write various sections of this article, and synthesize the key points for discussion. In total, 12 members were involved in a multidisciplinary setting including adult and pediatric gastroenterologists, IBD expert dietitians, and an IBD expert psychologist from 7 different countries. All authors then met in Tel-Aviv, Israel, in March 2023 over 2 days to discuss and agree on the topics included in this article. Given the lack of evidence for most of the included aspects, we provide an expert opinion synopsis, based on the best available literature, including published articles and key abstracts presented in conferences. Following the meeting, the lead authors (R.S.B., E.W.) structured the article, and all authors reviewed and approved the final version of the article. More details on the methodology appears in Supplementary Material online.

## Mechanism of Action of CDED

The CDED is a standardized diet consisting of 3 phases. The first phase (weeks 0-6) is highly restrictive, excluding all potential triggering ingredients, while emphasizing consumption of high-quality protein sources and microbiome-enhancing ingredients. The diet is liberalized for weeks 6-12 (phase 2), enabling a gradual introduction of previously restricted components. The third maintenance phase follows from week 13 for at least 9 months, until a more personalized approach is established. This phased approach facilitates ease of adherence for patients, making the CDED more manageable, allowing for better long-term compliance.

The design of the CDED was driven by avoidance of foods or food additives negatively impacting the microbiome and barrier integrity, which has recently been shown in the GEM cohort study to be a critical preclinical marker of disease susceptibility.^18,19^ Several foods have been demonstrated to impact the complex regulation of permeability (also influenced by mucus integrity, microbiome alterations, and inflammatory changes in the epithelium). Given this complexity, it has been difficult to apply reductionist approaches to test specific negative determinants of barrier integrity. Nevertheless, an improvement in barrier integrity was seen in a subset of CDED patients^14^ and is associated with a unique microbiome signature.^20^ Microbiome investigations have shown that reduction in Proteobacteria is associated with remission (and that nonresponders do not display this drop in Proteobacteria).^14^ It is important to note that microbiome changes are not fully corrected, even when clinical remission is achieved at 6 weeks. Exclusive enteral nutrition patients who resume a normal diet tend to lose some of the gains in dysbiosis correction (notably Proteobacteria increase), as *Escherichia coli* continues to be more abundant, even when sustained remission is achieved. Whole-community level microbiome studies have shown that the expansion of Firmicutes is present in both EEN and CDED but of secondary importance to the reduction in Proteobacteria when it comes to achieving remission.^12^ While EEN has not been shown to bring the microbiome closer to what would be considered a “normal microbiome” (in fact, lower diversity has been reported), CDED could potentially achieve this, as reflected in the persistent reduction in Proteobacteria and increase in Firmicutes.^13^ However, as defining normalization of the microbiome composition is challenging, following the function of this community is probably more relevant. This is consistent with data showing that short-chain fatty acid (SCFA) levels (at least in feces) are not associated with obtaining remission.^12^ However, the capacity to generate SCFA (ie, Firmicutes) and a reduction of consumption by other microbiome members (eg, Proteobacteria) is consistent with a metabolic profile that changes with successful dietary therapy towards health.^12^ Excessive primary bile acids are a marker of dysbiosis, but the most common secondary bile acids were not clearly associated with achieving remission through CDED.^12^

**Figure 1.**
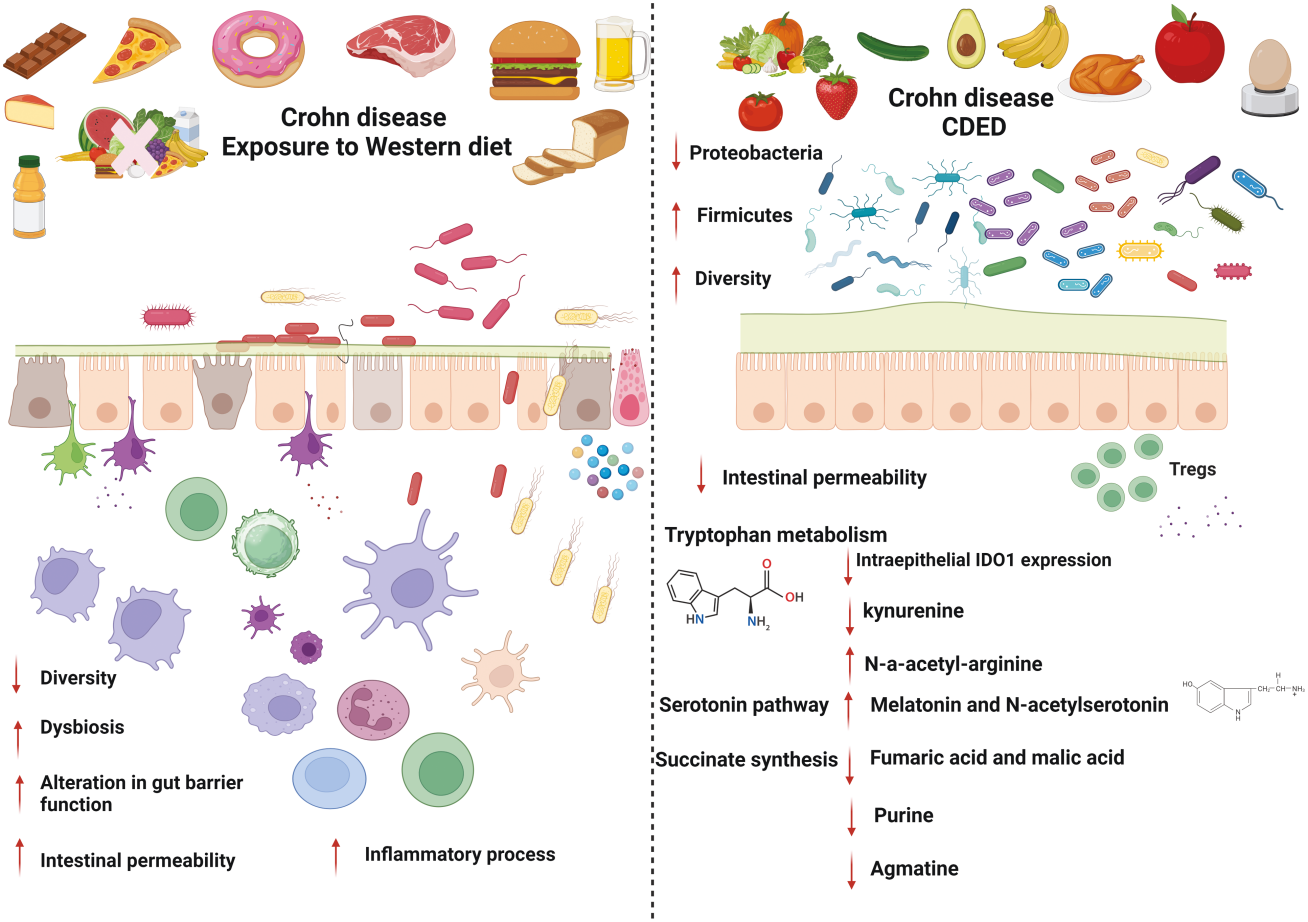
Understanding the role of diet in Crohn’s disease pathology: insights from available data and mechanistic implications for CDED.

A broader metabolomic screen showed that CDED + PEN- and EEN-induced remission was associated with significant changes in IBD-associated metabolites, such as kynurenine, ceramides, amino acids, and others. Sustained remission with CDED + PEN, but not EEN, was associated with persistent changes in metabolites.^10,11^ Interestingly, in mild to moderate pediatric CD, sustained diet-induced remission by both CDED + PEN and EEN is associated with a marked decrease in fecal kynurenine levels. Importantly, in samples from patients failing to sustain remission, no changes were observed. The reduction in specific kynurenine pathway compounds and the increase in serotonin pathway compounds are associated with diet-induced and sustained remission. This suggests a link between clinical outcome of dietary therapy and changes in the balance of tryptophan metabolism pathways between indole generation (microbiome-driven), kynurenine production (Indoleamine 2,3-dioxygenase 1 [IDO1]) enzyme-dependent (ie, host), and serotonin metabolism.^10,11,21^ Key mechanisms for the action of CDED are presented in Figure 1.

## Review of Published Evidence for CDED

### Pediatric Studies

The first experience with CDED started in children, while searching for a feasible alternative for those who could not tolerate EEN. Sigall Boneh et al reported on the first 47 patients who used this strategy to reduce inflammation among children and young adults with mild to moderate CD. After 6 weeks, 70% experienced clinical remission accompanied with reduction in Pediatric Crohn’s Disease Activity Index (PCDAI) and normalization of C-reactive protein (CRP).^22^ A pivotal multinational randomized controlled trial (RCT) was published a few years later, comparing CDED + Partial Enteral Nutrition (PEN) to EEN in children with mild to moderate CD.^14^ The CDED + PEN vs EEN showed superior tolerability and equal effectiveness in inducing remission at week 6, with response rates around 85%. Additionally, CDED + PEN led to significantly higher rates of sustained remission at week 12 compared with EEN for 6 weeks followed by a return to free diet.^14^ Remission achieved with nutritional therapy was associated with a marked decrease in Proteobacteria and an expansion of Firmicutes.^12,14^ However, by week 12, some patients still displayed an increased abundance of disease-associated *E coli*.^12^ In a follow-up study among children who achieved remission with both CDED + PEN or EEN by week 6, more than 80% were already in remission by week 3, suggesting that there is a large subgroup of pediatric patients with mild to moderate CD with a dietary-responsive phenotype that can be identified early in the course of therapy.^24^ Dietary responsive patients should be identified (similar to the commonly used term of “steroid-responsive,” etc.), especially given that this study achieved a high response (83%) and remission rates (64%) with both dietary treatments within the first 3 weeks. Defining patients as diet-responsive can impower them to follow dietary therapy for the long term or may reduce the burden of unnecessary restrictions. Importantly, compliance with dietary instructions was crucial to achieve response, and these studies demonstrated that CDED + PEN could address the adherence challenges that were often seen with EEN. Identification of patients with and without a rapid response to diet might help identify those who, with compliance, will achieve clinical remission by week 6 and 12 of the diet.^24^

Recently, the effect of CDED + PEN on fecal calprotectin (FCP; marker of mucosal inflammation) was retrospectively investigated in a group of 48 children with active CD and increased FCP levels (>250 µg/g), showing that a 12-week course of CDED + PEN treatment led to a significant decrease in FCP in the studied group and to normalization of this parameter in every third patient.^16^ Thirty-five percent of patients normalized FCP and 56.2% showed a 50% reduction of its values. All patients with normal FCP at week 12 were in clinical remission, and 94% of them had also normal CRP levels.

Niseteo et al communicated their experience with CDED compared with EEN in a group of 61 pediatric patients.^17^ Forty-one (all naïve to other therapies) had received EEN, and 20 (18 naïve, 2 after disease exacerbation) received CDED (with or without initial 2 weeks of EEN). Globally, 42 (68.9% of the patients) achieved remission, without differences between those who received EEN (65.9%) or CDED (75%). However, the group of CDED + PEN patients (with or without 2 weeks of EEN) showed significantly higher weight gain (*P* = .002) and increases in body mass index z-score (*P* = .001) compared with EEN alone. The benefit of using CDED + PEN as mono- or cotherapy with medications in children with mild to moderate CD was shown in a case series of 5 children who achieved sustained remission with this dietetic regimen.^26^ Together, these studies support the utility of CDED in mild-moderate pediatric CD.

A review of the published evidence for CDED is summarized in Table 1.

**Table 1.** Summary of CDED clinical studies and evidence.

Study	Study Population	Study Design	Intervention Group	Study Duration	Main Outcomes
**Pediatric Studies**					
Sigall-Boneh 2014^22^	Active CD patients from Israel, mostly mild to moderate disease. (*n* = 47; 13 were young adults)	Retrospective quasi-experiment	CDED plus 50% PEN for 6 weeks followed by CDED with 25% PEN for another 6 weeks, or CDED alone	12 weeks	Clinical response and remission were achieved in 79% and 71%, respectively, at week 6. CRP was normalized in 70%
Sigall-Boneh 2017^23^	CD patients with loss of response to biologics (*n* = 21; 11 were young adults)	Retrospective quasi-experiment	CDED plus 50% PEN for 6 weeks followed by CDED with 25% PEN for another 6 weeks, or CDED alone. Severe patients started with EEN for 2 weeks and continued with CDED plus PEN	12 weeks	Clinical response and remission were achieved in 90% and 62%, respectively, and lead to improvement in inflammatory markers at week 6
Levine 2019^14^	Mild to moderate luminal CD patients from Israel and Canada (*n *= 78)	RCT	Group1: CDED plus 50% PEN for 6 weeks followed by CDED with 25% PEN for another 6 weeks; Group2: EEN for 6 weeks followed by a free diet with 25% PEN for another 6 weeks	12 weeks	CDED plus PEN was better tolerated than EEN and led to 75% clinical remission vs 59% in the EEN group at week 6; CDED + PEN was superior to EEN group at 12 weeks in all clinical parameters
Sigall-Boneh 2021^24^	The same RCT^14^ cohort	Post hoc analysis	2 interventional groups as describe previously	12 weeks	Both groups (CDED and EEN) induced rapid responses (82% and 85% respectively) at week 3
Levine 2020^25^	Active CD from Israel (*n* = 4)	Case Series	CDED plus PEN	Up to 3 years	Case 1 and 2: CDED plus PEN effective monotherapy in uncomplicated mild-moderate CD disease, with > 1 year sustained remission recorded. Case 3: In penetrating disease, CDED plus PEN effective as a combination maintenance therapy post induction of remission with 8/52 EEN, antibiotics and anti-TNF. Resolution of fistula and perianal disease at 4 months repeat MRE. Case 4: CDED and PEN effective as rescue therapy in refractory patient. Regained response to biologics and sustained remission at 5 months.
Scarallo 2021^26^	Mild to moderate luminal-colonic CD patients from Italy	Cases series	CDED plus PEN or CDED alone	Up to 52 weeks	CDED with or withoutPEN presented a safe and effective therapeutic option asboth induction and maintenance monotherapy
Niseteo 2021^17^	Active CD patients from Croatia (*n* = 61)	Retrospective comparative study	2 interventional groups: EEN and CDED plus PEN. In the CDED plus PEN group, 80% of patients initially received EEN for 1-2 weeks.	6-8 weeks	CDED + PEN resulted in a 75% remission rate and was as effective as EEN in inducing remission and improved weight gain.
Matuszczyk 2022^16^	Mild-moderate CD patients with elevated FCP (*n* = 48) from Poland	Prospective quasi-experiment	CDED plus 50% PEN for 6 weeks followed by CDED with 25% PEN for another 6 weeks	12 weeks	FCP levels were normalized in 35%; 50% decrease in FCP in 54%
Stein 2022^27^	Remission CD (age 13-23 years) in Israel and USA (*n *= 18)	Prospective comparative study	Diets following withdrawal of immunomodulator or anti-TNF Group 1: CDED plus PEN Group 2: Free diet	52 weeks	No significant difference in 52-week remission rates (5/9; 55.6%) amongst CDED vs Free diet group (5/7; 71.4%), *P* = .63.
Martín-Masot 2023^28^	Mild- moderate CD (age 8-18 years) from Spain (*n *= 24)	Prospective to assess changes in dietary habits on CDED and compliance after 1 year	24 hour recall at baseline and 52 weeks on CDED	52 weeks	CDED resulted in reduction in the intake of ultra-processed foods (UPFs); a higher adherence to the Mediterranean diet (KIDMED score: 5 ± 2.1 at baseline vs 7.5 ± 1.4 at 52 weeks. After 52 weeks of CDED treatment, no patient had a very poor-quality diet compared with 33.33% at baseline.
María Clara Jijón Andrade 2023^29^	New onset mild to moderate and loss of response to biologics (age 10.7-15) from Spain (*n *= 15)	Retrospective quasi-experiment	CDED + PEN	24 Weeks	CDED + PEN among 15 patients with CD resulted in remission in all patients at week 6 and 12. Among them, 87% of treatment naïve patients-maintained remission at week 24 compared with 67% in patients who lost response to biologics. In treatment naïve patients the FCP and albumin improved at week 6, week 12, and week 24 (*P* < .05). Whereas in patients who loss response to biologics the reduction in FCP did not reach a level of significant.
**Adult Studies**					
Szczubelek 2021^30^	Adults with mild—severe CD CDAI > 150 (*n* = 32) from Poland	Real world evidence	CDED plus 50% PEN for 6 weeks followed by CDED with 25% PEN for another 6 weeks;	12 weeks	Clinical remission was obtained in 76.7% patients after 6 weeks and in 82.1% after 12 weeks of CDED. FCP improved vs baseline (*P* = .021).
Yanai 2022^15^	Adults (18-55 years) with mild—moderate CD (HBI 5-14) from Israel (*n *= 44)	Open-label, pilot randomised trial	Group 1: CDED plus partial enteral nutrition Group 2: CDED alone for 24 weeks.	24 weeks	At week 6, CDED + PEN resulted in 68% remission rate and CDED alone resulted in 57% clinical remission. 80% of those in remission at week 6 sustained remission at week 24.
Fliss Isakov 2023^31^	Adults (>18 years) with active pouchitis: cPDAI > 2 and mPDAI ≥ 5 (*n* = 8) from Israel	Nonrandomized, noncontrolled, open-label, Interventional Pilot Study	CDED plus 50% PEN for 6 weeks followed by CDED with 25% PEN for another 6 weeks	12 weeks	Clinical remission (cPDAI subscore 2) was achieved by 66.7%, 60%, and 46.7% of patients at Weeks 6, 12, and 24, respectively.

Abbreviations: RCT, randomized controlled trial; CDED, Crohn’s disease exclusion diet; PEN, partial enteral nutrition; EEN, exclusive enteral nutrition, CDAI: Crohn’s disease activity index, cPDAI: clinical Pouchitis Disease Activity Index IBDQ: IBD quality of life, mPDAI modified Pouchitis Disease Activity Index: HBI: Harvey Bradshaw Index; FCP-Fecal calprotectin.

### Studies in Adults

The success of CDED among children opened a new era and motivated investigation into dietary therapy among adult patients who usually did not accept an exclusive liquid diet (EEN). In a prospective pilot study, CDED+/- PEN induced clinical remission in 25 of 40 (63%) of adult patients with active mild-moderate CD at week 6, and sustained remission in 20 of 40 (50%) at week 24. Additionally, this strategy improved biomarkers with a significant decline in CRP and FCP concentrations at week 12. Lastly, endoscopic remission was observed in 14 of 40 (35%) patients in the intention-to-treat analysis by week 24 (with SES-CD score ≤3). Notably, the addition of PEN to CDED did not significantly improve outcomes during the first 12 weeks, although the number of patients in the CDED + PEN group who achieved sustained remission and gained weight at week 24 was numerically higher (60%) than that for patients in the CDED alone group (48%); this study was not powered to assess the differences between CDED + PEN to CDED alone.^15^ The combination of CDED + PEN also demonstrated efficacy in a small group of adult patients with CD who failed biological therapy despite dose escalation: 13 of 21(61.9%) of patients were able to recapture clinical remission after 6 weeks of therapy.^23^ A group from Poland described their experience with the CDED + PEN in a prospective study among 32 adult patients with active CD. Clinical remission was obtained in 76.7% of patients after 6 weeks and in 82.1% after 12 weeks of therapy, with significant improvement in FCP levels at week 12 compared with baseline (*P* = .02).^30^ Interestingly, CDED + PEN was recently reported as a successful sole therapy in a case report of a woman diagnosed with CD during pregnancy; the diet was sustained from week 14 of gestation until after delivery.^32^ However, extreme caution needs to be practiced when using restrictive diets in this population, and patients must be carefully monitored by medical stuff.

### CDED for Long-term Maintenance of Remission

Published evidence for CDED as a sole maintenance therapy for IBD is limited. The first article by Levine et al (2) describes 2 case reports whereby CDED and PEN was used as monotherapy to induce and sustain remission.

The study by Yanai et al supports the use of CDED as sole maintenance therapy in adults.^15^ In this randomized pilot trial in biologic-naive adults (*n* = 44) with mild to moderate CD, 50% of the ITT population (*n* = 20) were in sustained remission at week 24 on CDED+/-PEN as monotherapy, and 80% of those who were in remission at week 6 (*n* = 25) were still in remission at week 24. Both articles suggest a potential strategy of utilizing diet as monotherapy for maintaining remission in some patients with mild to moderate CD. However, it should be noted that the current available evidence for a 24-week duration is limited.

In children, the DIETOMICS-CD trial investigated the long-term outcomes of CDED + PEN in RCT comparing 2 weeks of EEN followed by CDED + PEN over 24 weeks to 8 weeks of EEN followed by PEN with free diet. At week 24, 60% of patients from CDED + PEN and 42% from EEN group remained in clinical remission (*P* = .18). Additionally, among patients who achieved clinical remission at week 8, 78% from the CDED + PEN group and 64% from the EEN group maintained remission up to week 24 (*P* = .15). Importantly, the EEN group received more immunomodulators than CDED + PEN group (100% compared with 56%, respectively, *P* = .002).^33^

Clinical experience from the Tel Aviv Souraski Medical Center (N.M.) reports that despite the high response rate during the first stage, more than 80% of the patients stopped the diet by week 24, mostly due to compliance issues. Interestingly, even patients who did not make it through to CDED stage 3 reported significantly changes in their everyday diet and consumed less processed foods.^34^ Supporting the experience in Tel Aviv, recently a group from Málaga described their experience with 24 children who followed the CDED for 52 weeks. They reported an improvement in dietary habits, including a reduction in the intake of processed and ultra-processed foods and a higher adherence to the Mediterranean diet.^28^

Importantly, educating CD patients regarding CDED empowers them and gives them tools to help control their disease activity. An additional aspect to consider in maintaining remission is the appropriate timing to transition from a rigorous to more liberal diet. The involvement of trained dietitians becomes crucial in this context, as they possess valuable insights into patients’ habits, limitations, and potential development of negative relationships with food, including avoidant restrictive food intake disorder (ARFID). In certain cases, dietitians may guide patients towards a less restrictive diet and collaborate with psychologists to improve food-related behaviors. Ultimately, most patients will require drug therapy; long-term dietary monotherapy can be considered in highly motivated patient, with close follow-up; drug and diet should usually be combined in less motivated patients.

## Patient Selection: When to Use and When Not to Use CDED?

### “Ideal” Candidates for CDED

Mostly based on the original RCT, CDED has demonstrated its highest effectiveness in mild to moderate, uncomplicated, pediatric, luminal CD patients with a relatively short disease duration (ideally treatment-naïve), with active inflammation and generally ileal or ileocolonic disease^14^; more recent studies support similar indications in adult patients, as detailed previously.^15,30^ However, it is not clear whether CDED is beneficial for the therapy of severe and extensive disease, penetrating disease, perianal fistulae, and for extraintestinal manifestations, and whether it can be suggested to patients with strictures. These groups of patients have not been represented in most published trials, and evidence regarding these conditions is mostly based on case series and personal experience. Real world evidence (RWE) suggests that CDED can be clinically beneficial as an adjunctive, rescue, or “bridge” therapy in those with more severe disease.^34,35^

### CDED in Diverse Populations and Special Conditions

#### CDED in severe CD

Niseteo et al^17^ reported the use of EEN for 2 weeks prior to CDED + PEN in children with mild to severe CD, as described previously. Remission was achieved in all 3 patients with severe disease. The high remission rate was attributed to the pretreatment with EEN before CDED + PEN was introduced. In another real-world study, Szczubełek et al^30^ included 4 patients (12.5% of the cohort) with severe disease (CDAI >450). Three of these patients (75%) achieved clinical remission (CDAI <150) after week 12 of CDED.

Similarly, CDED was effective in 3 of 5 (60%) children with severe disease (PCDAI >40) who failed to achieve remission in response to biological treatment.^22^ In contrast, in a cohort study by Sigall-Boneh et al, 0 of 4 patients with Harvey Bradshaw index (HBI) ≥13 compared with 13 of 21 (76.4%) with less severe HBI scores achieved clinical remission. However, all patients in this cohort had failed biological therapy, and most had a long disease duration. Nevertheless, 90.4% of the patients did achieve clinical response by week 6 of the diet.^23^

Crohn’s disease exclusion diet was evaluated retrospectively in a RWE study^34^ with a diverse population of CD patients, among whom 48 patients initiated the diet due to clinically active disease. In this cohort, they reported remission in only 1 of 6 patients with a severe disease activity (HBI >13).

In addition, a case report by Scarallo et al^35^ described the use of CDED in 2 young boys with severe refractory disease, and Levine et al^25^ described its use as a maintenance diet following EEN for induction in a more severe case of penetrating disease whereby a 15-year-old male with an entero-enteric fistula received 8 weeks of EEN induction followed by CDED and PEN.

This sheds some light on the selection of patients qualifying for dietary treatment. As expected, severe disease is less amenable to CDED, but some patients do achieve clinical remission and most achieve at least clinical and biomarker response. Thus, in these patients, CDED can serve as a bridge intervention to support slower acting agents or potentially when immunosuppression is less favorable, such as in patients prior to surgery or in patients with septic complications/abscess.

An additional approach was established when patients were hospitalized or presented with severe relapse after losing response to biological therapy.^23^ A short course of EEN followed by CDED was assessed once initial improvement was established, or when patients were discharged from hospital: 3 of 5 (60%) achieved clinical remission at week 6. Following this successful initial experience, patients with more severe disease were studied in a prospective clinical study (DIETOMICS). The results of the entire cohort (currently published as an abstract at ECCO) revealed high remission rates with 23 of 30 (76%) from the CDED + PEN group and 14 of 26 (54%) from EEN group achieving clinical remission at week 8; *P* = .07. At week 14, steroid-free clinical remission was achieved in 21 of 30 (70%) in CDED + PEN and 16 of 26 in EEN (61.5%, *P* = .56), with significant improvement in CRP and FCP over time as well with higher use of IMM in the EEN group compared with the CDED + PEN (56% compared with 100% respectively, *P* = .002). Taken together, these studies suggest that while CDED may not be as effective in severe CD, it likely has a role in some patients, mostly as an adjuvant therapy or bridge before medication therapy is initiated. In addition, among the 10 CDED patients who did not use IMM at week 14, 90% remained in remission. Remarkably, 5 patients continued CDED phase 3 without immunomodulators until week 24, maintaining clinical remission. These findings indicate that diet-based monotherapy might be an additional strategy for inducing and sustaining remission in certain CD patients.^33^

#### CDED for perianal disease and extraintestinal manifestations

There are no publications regarding CDED for perianal disease or extraintestinal manifestations (EIMs). Unpublished RWE (by N.M.) reported use of CDED among 6 patients with perianal disease who were fairly compliant with the diet; 2 patients reported less pain and less discharge from the fistula. In the same cohort, 8 patients suffered from peripheral arthritis/arthralgia or from oral aphthae: 4 patients who had a good compliance to the diet reported an improvement of their EIMs. The other 4 patients who did not improve had a low or unknown compliance to the diet. Clearly, more evidence is required to support the potential role of CDED in these complicated population.

#### Using CDED for pouchitis

The similarities between the pathogenesis of pouchitis and CD has led Fliss et al to examine the effectiveness of CDED for pouchitis patients in a pilot study.^31^ Adult patients (*n* = 15) demonstrated a significant improvement in clinical measures; 10 of 15 (66.7%) achieved clinical remission (cPDAI subscore ≤2) by week 6, and 6 of 15 (40.0%) and 7 of 15 (46.7%) achieved clinical and endoscopic remission (cPDAI ≤2 and mPDAI <5) by weeks 12 and 24, respectively. Clinical and biomarker response were associated with adherence, and FCP improved significantly among patients with perfect adherence to diet at week 12. Furthermore, patients with more severe endoscopic disease were less likely to respond to the diet.

### CDED as an Adjuvant to Biologics

Existing literature on diet as a cotreatment is lacking. What is currently known is focused on PEN (partial formula-based diets) in combination with medication. A meta-analysis of 4 studies with a total of 342 patients found that biological therapy (infliximab) coupled with PEN, together with a low fat or regular diet was more likely to yield sustained remission at 1 year than biological therapy alone.^36^ However, limitations included small number of studies, most of which were retrospective, and all were from the same country. Sigall-Boneh et al analyzed the efficacy of CDED to induce remission in patients in whom anti-TNF treatment had failed as described previously.^7^

In a retrospective study, Jijon Andrade et al evaluated the effectiveness of CDED + PEN in 15 CD patients over 24 weeks, including 6 who had lost response to biologics. All patients achieved remission at week 6 and maintained it until week 12. At week 24, remission was maintained by 87% of treatment-naïve patients and 60% of patients who had lost response. Treatment-naïve patients showed significant reductions in FCP and improved albumin, while patients who had lost response showed a nonsignificant reduction in FCP. These findings suggest that CDED + PEN is more effective in treatment-naïve patients.^29^ Recently, a case series by Lionetti and colleagues^26^ demonstrated examples of CDED + PEN plus comedication in children with CD. These included CDED plus infliximab in a patient with luminal + perianal CD, CDED plus azathioprine in a patient newly diagnosed with severe CD, and CDED in the setting of biologic loss of response and biologic refractory disease (ustekinumab plus CDED). There were also examples of CDED as rescue therapy for refractory patients. Identifying nonpharmacological strategies to induce remission in patients whose disease is partially responding to medications, or refractory to conventional therapies, is an unmet need. Though further studies are needed, induction or reinduction of remission with CDED as an adjunct to pharmacologic therapy has many potential advantages including reducing exposure to further drugs while targeting the environmental mechanism of disease without additional toxicity, which could be especially important for children and adolescents with decades of life with the disease ahead. The identification of dietary-responsive patients early in the disease course might encourage the use of the CDED among patients who could benefit from reintroduction of dietary therapy when a secondary loss of response to other therapies occurs as diet could be used here before approaching a different optimization approach.

### When We Should Not Use CDED

**Figure 2.**
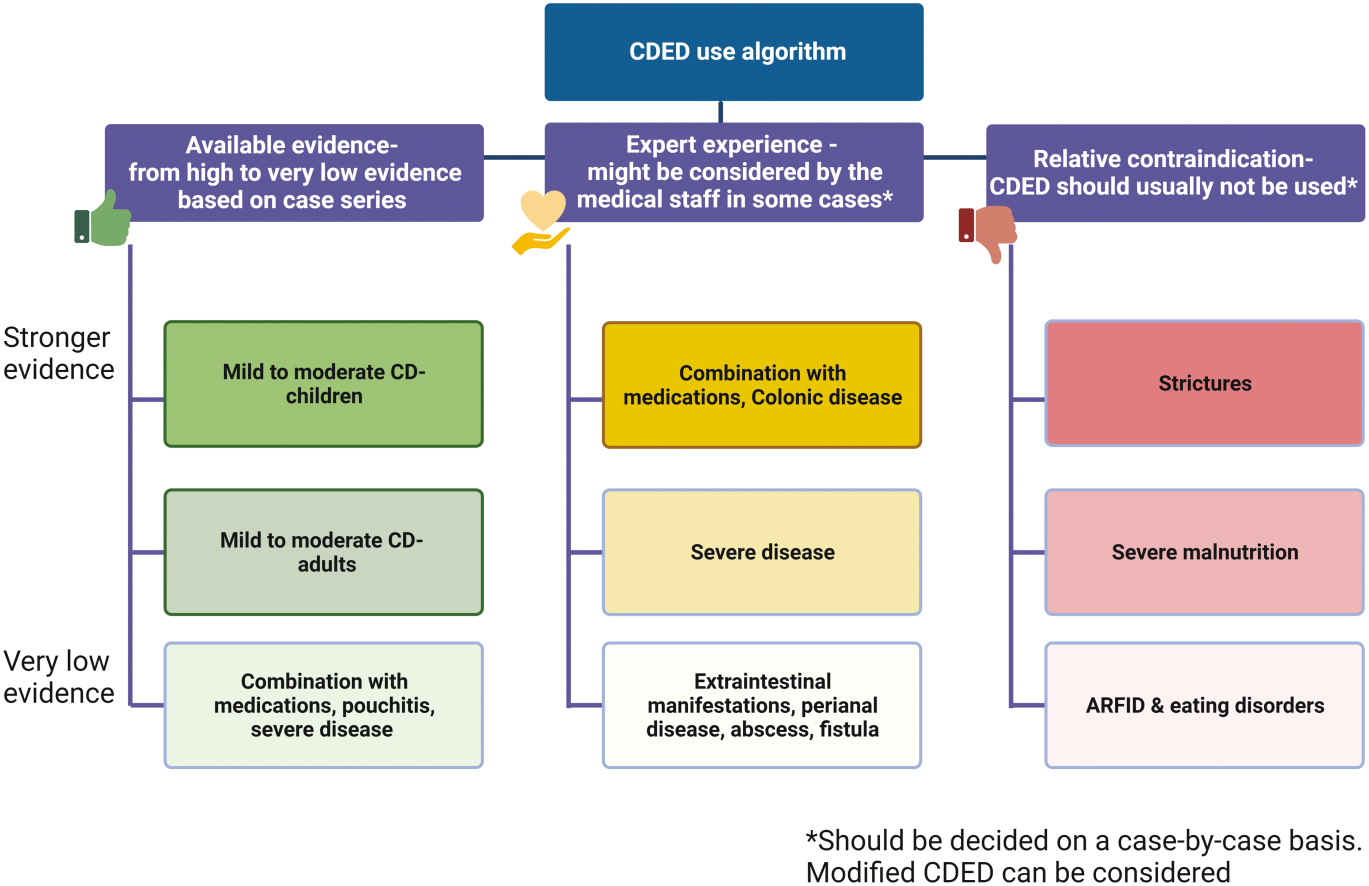
Optimizing CDED: suggested indications, contraindications, and essential considerations.

Figure 2 and Supplementary Table 1 serve as guides to when CDED might not be appropriate. Following index studies in pediatric IBD with strictly defined inclusion and exclusion criteria, there is inevitably a period of “mission creep” where patients showing inadequate response to other therapies (and/or facing surgery) are given the newer treatment, such as CDED. In contrast, historical and more recent data have shown that the judicious use of EEN can provide real benefit in terms of improving perioperative outcomes and avoiding surgery in some patients.^37,38^ In the index CDED-RCT,^14^ notable exclusion criteria were mainly left-side colonic involvement, active extraintestinal (eg, joint or liver) disease, active perianal disease, and prior surgery of complicated CD (stenosing/penetrating). Patients who lost response to biologics were reported to benefit from CDED + PEN, but notable exclusion criteria were fever >38.5°C, current bowel obstruction, intra-abdominal abscess, intercurrent or opportunistic infection.^23^ With increasing experience with CDED + PEN, we should continue to exercise great caution when using food-based enteral nutritional therapy in the presence of stenotic findings on imaging, and even more so if endoscopy has shown a stenotic segment or symptoms of stenosis are present. In the presence of mild stenosis on imaging, a period EEN may be attempted. Endoscopic stenosis or symptoms of stenosis at the time of considering EEN should trigger follow-up imaging to document resolution of prestenotic dilatation and resolution of clinical symptoms of stenosis before considering broadening the oral diet towards CDED + PEN phase 1. A fixed stricture should prompt timely consideration of surgery and preoperative nutritional optimization with EEN or, when this is not tolerated, total parenteral nutrition (TPN) in keeping with ESPGHAN guidelines.^39^

Before prescribing CDED, several patient-related factors must first be considered. These include patient’s history of maladaptive eating behaviors and eating disorders, including ARFID, access to recommended foods, ability to maintain the cost of the diet, religious and personal practices, and impact of diet on quality of life.^40^ Other important aspects to consider are current psychopathology and overall motivation to adhere to the diet, as discussed in further detail later on.

## Potential Pitfalls and Solutions

### Starting CDED at Different Phases

The decision not to start at phase 1 should be on a case-by-case basis, but all the evidence to date supports starting at phase 1, especially for newly diagnosed patients; this also allows for identification of dietary-responsive patients. In several cases, patients are willing to start the diet for prevention while they already obtained remission with either EEN or medication. In addition, patients on biological therapy who are willing to start the diet can probably start the diet as adjunctive therapy, also considering the possibility to start preferably from phase 2, or even 3 with support from a dietitian. Other, similar diets, including the Mediterranean diet, can also be considered in this setting. In patients who have successfully completed a course of EEN, commencement on the maintenance phase to prevent rebound dysbiosis may be considered, but this has not been tested.

### Reinduction of CDED Phase 1

Some limited published experience shows that in patients who flare after transitioning to the next phase, remission can be regained by reverting to the previous phase. Dietary and disease activity assessment using HBI or PCDAI, FCP, and CRP, could be helpful to determine if the patient is flaring and the severity of the situation, or if indeed symptoms are due to an intolerance, for example, to the increased fiber intake in phase 2. This can be problematic in patients with luminal narrowing and counseling regarding the texture and portions consumed per meal need to be discussed. If the patient is flaring because they have deviated from the diet, then a shorter return to phase 1 or 2 for 2 to 4 weeks as described by Levine et al,^25^ followed by a return to the maintenance phase, may be sufficient to regain remission. In more severe flares or if a patient has deviated for a prolonged period, a complete return to phase 1 or 2 may be required to regain remission and to correct the dysbiosis. In general, it appears that patients who have responded to CDED in the past (diet-responsive phenotype) will do so again. Recurrent flares or CDED dependence require assessment of disease and may warrant an adjunctive therapy, dose escalation, or drug change if they are on CDED as monotherapy.

## Psychological Consideration and Other Challenges

### Psychological Effects of Elimination Diets

Previous studies have found that IBD patients frequently use food restrictions and exclusions to control their symptoms and, as a result, report lower psychological well-being and many emotional challenges such as socializing, taking part in special occasions, fear of an adverse event or disease relapse, and enjoying food.^41-45^ Food-related quality of life (FRQoL) was found to be impaired among IBD patients and associated with lower intake of key nutrients.^46^ Other patients, however, may find it comforting knowing what they are allowed and not allowed to eat, to the point that they eventually develop a kind of “fanaticism” towards the chosen diet. This extreme adherence and restriction may increase anxiety and for some even the risk of developing an eating disorder, especially in adolescents.^40^

Therefore, comprehensive dietary assessment and monitoring are crucial in patients that consult for CDED to ensure that the restriction will not prolong unnecessarily and to prevent a routine of restrictive eating behavior. Avoidant restrictive food intake disorder is a type of eating disorder with symptoms that are commonly seen in CD patients, especially pediatric, which include abdominal pain, nausea, “picky eating,” dysphagia, delayed gastric emptying, and weight loss. As opposed to different types of eating disorders, ARFID influences dietary intake for reasons that are not related to body image such as perception of pain, fear of negative reaction, and lack of interest in eating.^47-49^ Exclusion diets in CD might increase the fear of food, as found in other chronic illnesses.^50^ It is important to screen patients (and parents, if appropriate) to identify patients/parents with high anxiety that might affect the development of a negative relationships with food that might lead to ARFID.^51^

The effect of diet on QoL also needs to be considered while consulting for CDED. Interviews held with CD patients revealed an impact on QoL, indicating that limiting food choices and being on a restrictive diet negatively influence the enjoyment from food, as well as increase the fear of an adverse event or disease relapse.^42,45^ The resulted effect of poorer FRQoL was associated with lower intake of key nutrients from a comprehensive database collected from 1221 patients with IBD.^46^ On the other hand, in some cases, patients reported an improvement in QOL, likely related to reduction in inflammation and improvement of symptoms with CDED, as described by Szczubełek et al who showed that after 6 and 12 weeks on CDED, adult patients reported an improvement in QOL using IBDQ.^30^

### Global Consideration for CDED

One remaining challenge is adjusting CDED to different countries/regions. This is especially important for diet-based interventions, since both the sources/types of foods and especially the eating habits vary globally. The CDED was initially designed based on epidemiologic data from “Western countries,” such as EPIC and the US Nurses cohort, as well as the familiarity of investigators with dietary options from these countries. As use of CDED has grown, so has interest in various regions (eg, Asia) and the need for local adaptations. The current published literature includes experience from mostly Europe, Israel, and Canada.^14,16,17,30,34^ Unfortunately at this time, there is neither clear evidence regarding variability in success of the diet across the globe nor guidance on how to adjust the diet, but we hope to at least raise awareness and offer some ideas on how to move forward. In our experience, this situation has not posed complications with patients immigrating from different regions since there is great versatility of the recommended and allowed foods. Additionally, a large variety of condiments for cooking can be used to adjust recipes accordingly. An additional advantage that could help expand the use of CDED in different geographical locations and cultures is the availability of CDED information, including handouts, recipes, and meal plans in 10 different languages in a dedicated mobile app.

One aspect to consider is differences in eating culture. This has been described for EEN, for example, where clear geographical differences in use, acceptance, cost/coverage, and barriers have been reported.^5,52^ For CDED, this would apply to both the restricted foods and the list of allowed foods. To adjust for this, collaboration with local experts is advised, where following the principals of CDED with local adjustments is needed, together with research to assess difference in effectiveness. Differences in the same foods in different regions have been described, as certainly the amount of processing varies dramatically. Finally, food security and cost remain major barriers in many countries, making any dietary intervention more challenging, since ultraprocessed foods remain cheaper and more available.

### Special Considerations: Vegetarians, Vegans, and Allergies

Evidence on CDED among individuals with different types of dietary habits such as vegetarian, vegan, and allergies is scarce and is mostly based on practice and personal experience. The main concern with following a vegetarian and, even more so, a vegan version of CDED is ensuring enough protein and vitamins (eg, B12), especially in children, to allow for normal growth. The CDED contains recommended protein sources such as chicken breast and eggs. The role of dietitians in these patients is pivotal since dietitians should calculate and adjust the protein intake and allow for substitute alternatives to ensure sufficient consumption. According to recent ESPEN guidelines, adults patients are required to consume 1.2 to 1.5 g/kg of protein during active disease and 1 g/kg during remission.^7^ This amount could be met by addition of formula, concentrating the formula up to 1.5 Kcal/mL, addition of egg whites, and consumption of fish when patients agree, up to 2 to 3 times per week until the desired intake is met. In vegans, there are fewer options available, and closer monitoring is imperative. Patients who are willing to follow CDED should be aware that several compromises need to be made to meet the requirements; a plant-based protein formula could be used. Achieving the requirements with whole foods rather than protein supplementation is preferred, as many supplements contain additives. Consumption of legumes during active disease could be challenging due to the potential symptoms these might cause; this is why legumes are integrated into phase 2 after clinical improvement is achieved. In some anecdotal cases, where patients are used to consuming these foods, the dietitian could use legumes as a protein source or recommend a texture modification such as a homemade orange lentil paste or hummus. Additional protein sources could include a free additive protein enriched yogurt, according to the dietitian discretion. Adjustments for allergies are based on the dietitians’ guidance to meet the protein requirements, as described previously.

## Role of Dietitian and Nutritional Management

The dietitian’s role in the multidisciplinary team (MDT) has been recognized for many years.^53^ The role of dietitians in the management of IBD patients has evolved over the last decade to a leading role in guiding the provision of therapeutic diets, as summarized by Fitzpatrick et al.^1^ Dietitians are responsible for providing the dietary instructions of CDED in clinical practice and research.^54^ Dietitians will complete a full nutritional history assessment to understand the patient’s dietary habits, dietary preferences, lifestyle, and potential deficiencies or risk for undernutrition. In clinical practice, the role of dietitians is to adjust the patient’s diet according to the CDED principles and to make sure patients can and will consume the diet according to the instructions.

The best resources that a dietitian can provide to patients and their families is time and dedication to explain the basis of the treatment, as well as transmitting encouragement and motivation through the different phases of the diet. This treatment needs continued support and monitoring to ensure that dietary compliance and requirements are met. It is important that patients feel that they have someone to turn to when doubts or difficulties arise through the process, so having a support system composed by different professionals (MDT) and the patient’s environment is crucial to achieve success.^55^

As patients in many cases express hope that the restrictions will not last forever, it is important to emphasize that this is a temporary phase, and they will return to consume foods that they like, but maybe less frequently. Dietitians should guide patients on how to have a healthier dietary lifestyle and importantly, give them a sense of balance, which will allow them to maintain the diet for the long term.

Dietitians can help with cooking tips, recipes, advice for events, eating out, vacations, and so on. In the CDED studies, dietitians played an important role with analyzing the patients’ compliance using food diaries and a dedicated questionnaire to assess compliance with additional Likert scale assessment, based on the dietitian judgement, which makes the dietitian’s role mandatory in conducting clinical trials.

Therefore, the dietitian’s role is fundamental, and the differences between just providing the diet handout to a more individual approach can change the adherence to diet completely, which will improve the success of dietary therapy.

### Personalization of CDED

It is challenging to design a diet that would fit all patients. In some cases, there are gray areas where it is unclear whether specific foods are appropriate for individual patients. Dietitians should provide patients with knowledge on how to read food labeling and choose their foods wisely, adjusted to the phase they are in. It is important to recognize that the diet was designed with recommendation to consume several “mandatory foods” that were chosen for nutritional completeness reasons, not directly inherent to the dietary therapy. For these reasons, some adjustments/alternatives can be considered, and we now prefer the term *recommended foods*, not *mandatory*. Chicken breast and eggs were chosen to guarantee enough protein; however, if the patient will not eat eggs or chicken, the dietitian can provide alternatives and solutions as mentioned previously. The same applies to potatoes, bananas, and apples. These foods are recommended to increase consumption of resistant starch in order to produce more SCFAs and pectin. In cases where patients cannot tolerate these food items or do not want to consume them, the dietitian should provide substitutes using different fruits and vegetables based on the patient’s personalized tolerance and reduce the pressure from the concept of mandatory foods.

Regarding fruits and vegetables, the dietitian should guide the patients based on their individual tolerance to fiber. In cases with concern for strictures, the dietitian might recommend different textures to help with tolerance. In contrast, if patients can tolerate specific foods that do not appear in the general handout, the dietitian can guide and recommend a more tailored approach.

**Figure 3.**
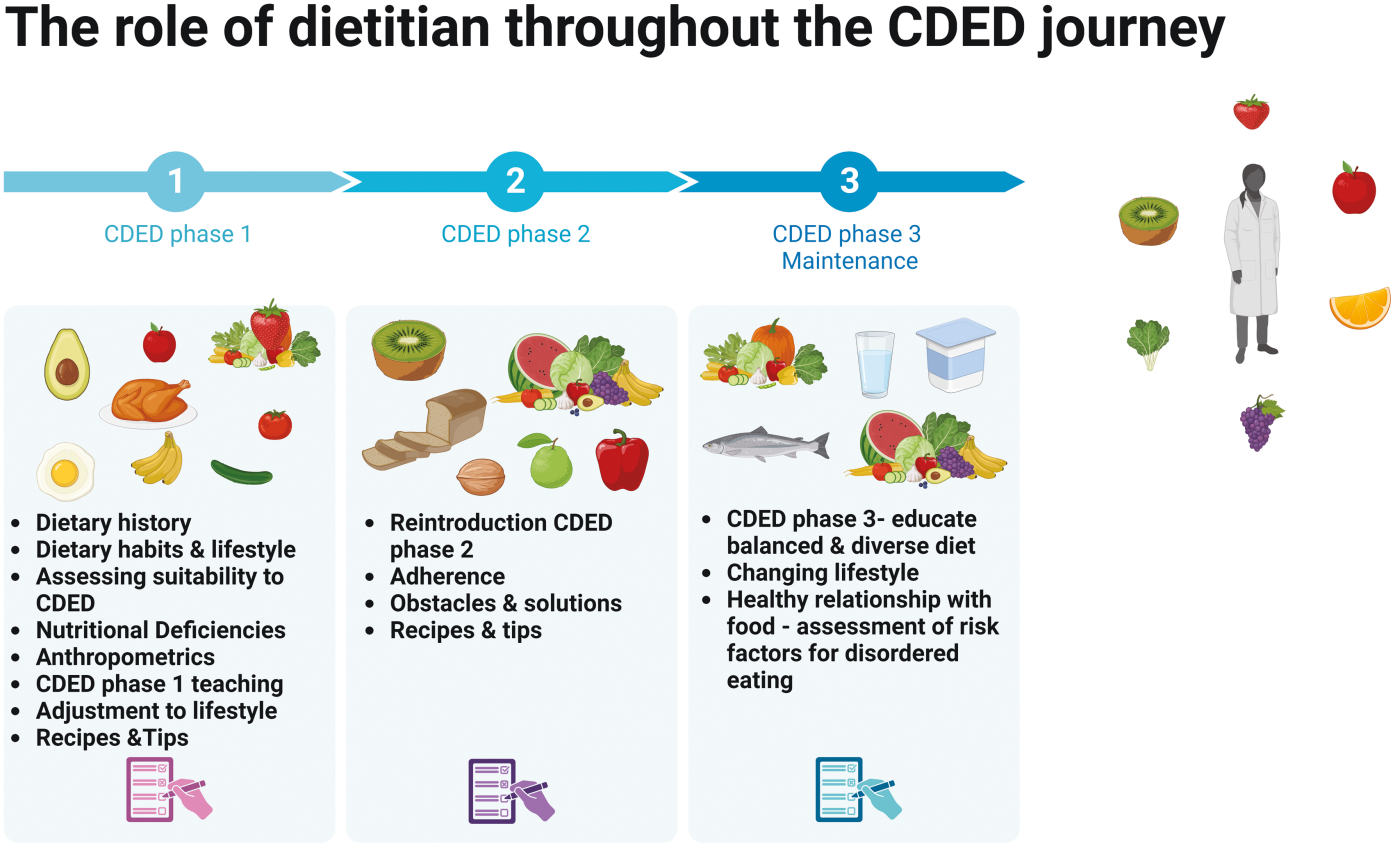
Empowering health through expert guidance: the crucial contribution of dietitians throughout the CDED phases.

In addition, in cases where patients are suffering from irritable bowel syndrom symptoms, the dietitian will guide them to restrict foods (included in the CDED) that might trigger their GI symptoms that are high in fermentable sugars (as listed in the FODMAP diet [such as apples, garlic, onions, etc.]).^56,57^ Dietitians should guide patients through the different phases of the diet. The most challenging phase is the third, when understanding the concept of balancing the diet is critical. Even though the diet was designed as a general standardized restrictive diet, as we gain more experience, adjustments are made to accommodate patient preferences and maintain long-term adherence to the diet. Figure 3 illustrates the dietitian’s role in guiding patients through the various CDED phases.

## Practice Points and Practical Considerations

### Educational and Support Resources

Patients commencing CDED should be supported by a dietitian or other clinician trained to use CDED. Patients should be supported by receiving a comprehensive list of allowed and disallowed foods, in addition to meal plans and recipes. Useful tools such as apps can support patients and families whilst following CDED; these could include sweet and savory recipes in addition to videos and tips on how to prepare CDED-compatible, stage-specific foods. Additionally, resources including shopping lists, FAQs, a support line, and the capability to record food consumed are available in smartphone apps, which can then be viewed by their dietitian or clinician to assist with diet compliance and nutritional adequacy. To our knowledge, the only existing CDED educational resource, which also offers patient support as detailed previously (including >200 CDED-based recipes) is the Modulife App, which is available in 10 different languages with reference to local foods and customs. Besides the patients’ app, the Modulifexpert.com platform offers free access to a detailed training course and other supportive resources on CDED for practitioners; any healthcare professional intending to use CDED should consider undergoing such training and keep updated on developments with CDED using such resources and the emerging scientific literature.

### CDED Limitations: Diet Cost and Time Considerations

Cost of food is a considerable factor for many families, particularly in the current cost of living crisis. Indeed, price is a major barrier to maintaining a healthy diet, even for the general population, but Herrador-Lopez et al^55^ analyzed the cost of food included at each of the CDED phases and concluded that it does not cost more than the average spent by a Spaniard on their groceries and is cheaper than EEN. If the patient or their family cannot afford formula, CDED and a calcium supplement could be an effective alternative for certain patients.^15^

The time available to spend in the kitchen by parents or caregivers can undoubtedly be one of the limiting factors for CDED. It is important to take into consideration that some families may prefer some degree of freedom in making dietary choices, while others may require more specific guidance. If required, a personalized meal plan can be prepared for patients based on food preferences and dietary needs.

### Assessment of Diet Compliance

Compliance is an important consideration when prescribing dietary therapy, as high adherence to therapy was associated with achieving clinical remission in both an index RCT^14^ and a retrospective cohort study, while patient or disease characteristics were not.^34^ Compliance can be monitored via dietary recall or a food diary completed by patients and assessed by dietitians at regular intervals throughout the dietary therapy. Additionally, use of the modified medication adherence report scale (MARS) questionnaire was utilized to measure compliance by Levine and Yanai et al.^14,15^ There are several ways to increase compliance, and dietitians have a pivotal role in both assessing compliance and improving it.

## Summary and Conclusions

The first report on CDED was published about 10 years ago, but since then multiple publications have enriched the current understanding of the mechanisms, best uses, challenges, limitations, and opportunities of this unique therapy. While high-quality evidence has shown clear benefits for the use of CDED to induce remission in children and adults with mild-moderate luminal CD through RCTs, we have included literature supporting CDED use in additional situations. It is important to bear in mind that this field is constantly evolving, and consequently, the principles of exclusion may also evolve over time, necessitating adjustments to the diet accordingly. For instance, recent studies suggest that not all emulsifiers have an equivalent negative effect,^58,59^ but further investigation is warranted to ascertain their true impact.

**Figure 4.**
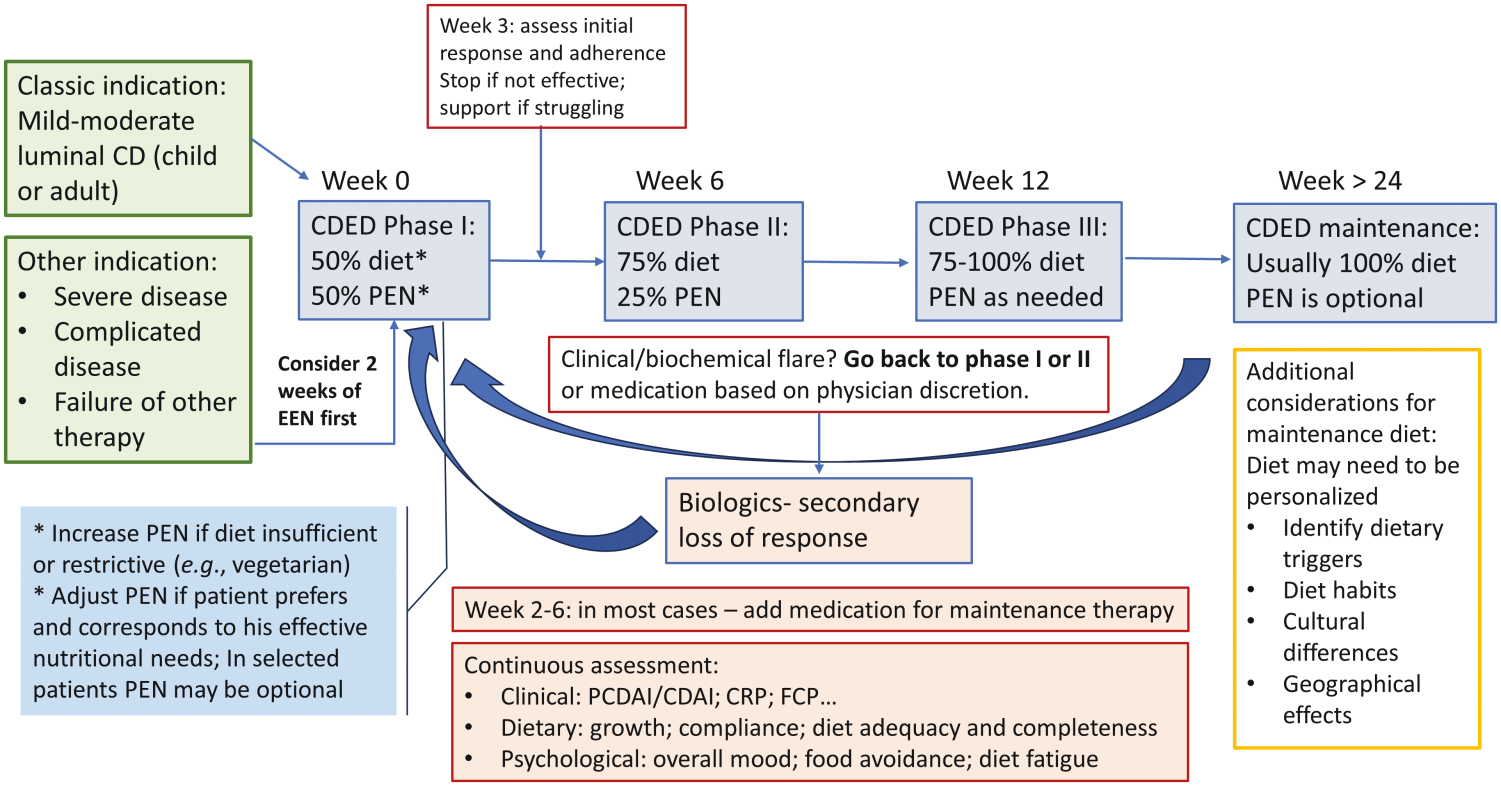
Navigating CDED: an algorithm for implementing and monitoring CDED in Crohn’s disease management.

**Figure 5.**
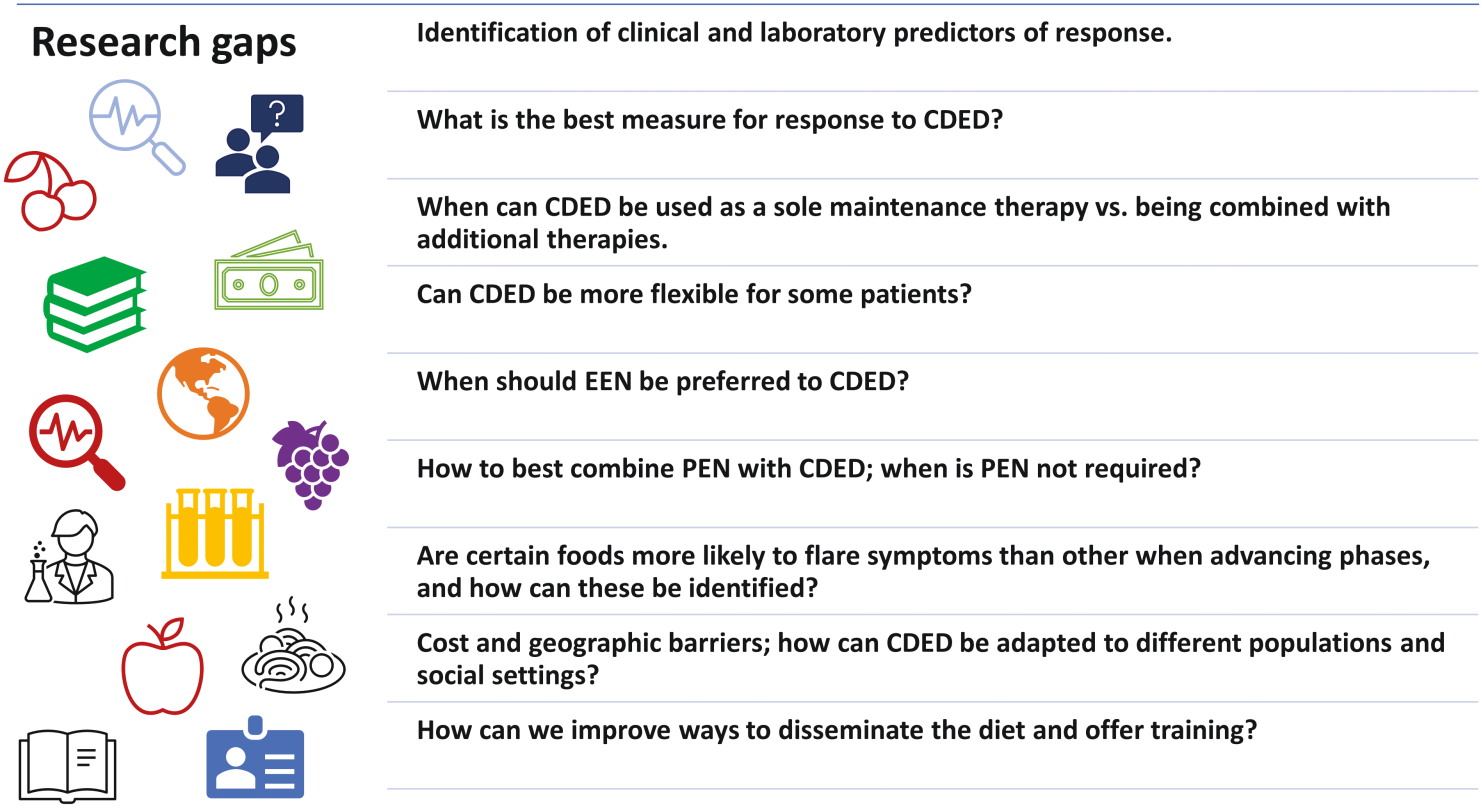
Research gaps and future opportunities for CDED.

Some of the practice will likely require individualized care, guided by the cumulative experience of physicians and dietitians. We hope that through this article we were able to share some of our experience and insights and have presented these principles in Figure 4. Some of the most important messages for users of CDED are the need for a multidisciplinary supportive team, appropriate training and patient support, close follow-up of patients, and awareness of the nutritional and psychological challenges and solutions. The directions for future research and development we identified are diverse and include some important topics, as presented in Figure 5. We hope that this article will serve as a resource for those treating patients using CDED and will stimulate further research and discussion.

## Supplementary Data

Supplementary data is available at *Inflammatory Bowel Diseases* online.

izad255_suppl_Supplementary_Tables_1
