# Supplementary material for: The Crohn’s Disease Exclusion Diet: A Comprehensive Review of Evidence, Implementation Strategies, Practical Guidance, and Future Directions
Source: Inflamm Bowel Dis. 2023 Nov 18;30(10):1888–902. doi: 10.1093/ibd/izad255 (PMC11446999; doi:10.1093/ibd/izad255)
Supplement: izad255_suppl_Supplementary_Tables_1 [file izad255_suppl_supplementary_tables_1.docx]

**Supplementary Methods section:**

‘A group leader was assigned for each topic. Each group conducted the literature search based on their team leader's instructions, who guided them and gathered the relevant information. Given the paucity of published data and the need for personal experience for some of the topics, we also relied on the expertise of each member and the group as a whole to identify and discuss all topics. Subsequently, once the initial drafts were completed, E.W and R.S.B, merged all the collected data to create a unified manuscript circulated among the entire team for a collaborative discussion through emails and virtual meetings, prior to the in-person meeting. Each group leader was tasked with preparing several slides for the meeting, presenting information related to their respective sections, including an overview of the existing evidence, highlighting points for discussion within the group, and identification of research gaps.

We thoroughly examined the compiled data during our discussion session, fostering a constructive dialogue among team members. Conclusions were drawn based on the insights and consensus reached during these discussions, and these conclusions were meticulously recorded. Following the meeting, E.W and R.S.B incorporated the conclusions reached and revised the manuscript accordingly. This revised draft was then shared with the entire team for further input and review until a final version was agreed upon.’

**Supplementary table 1: Suggested indications and contraindications for using CDED**

| **Factors to consider** | **Ideal CDED candidates** | **Possible candidates for CDED** | **Inappropriate candidates for CDED** | **Factors to follow while on CDED** | **Modifications to consider** |
| --- | --- | --- | --- | --- | --- |
| Diagnosis and disease characteristics | - Active mild-to-moderate luminal CD children and adults Patients - Patients with mostly ileal involvement | - Insufficient evidence: - Isolated colonic CD - Severe cases - Complications including fistulae, abscess, strictures, perianal disease, and bowel resections - Pouchitis | - IBD in remission with IBS-like symptoms - Ulcerative colitis | - Diet adherence - Response by activity index and inflammatory markers (CRP, ESR) by week 3 - Clinical remission by week 6 - Improvement in FCP at week 12 | - Increase PEN in case of malnutrition or difficulties in consuming recommended foods |
| Diet and lifestyle history | - Carnivores/omnivores with motivation to adhere to the diet | • Vegetarian or  Vegan | - Patients/families without motivation to follow the diet - Poor ability to adhere to the diet (*e.g*., support, financial) - Concern for diet impairment of QoL | - Adherence - Family dynamic with pediatric patients | - Increase PEN in case of vegetarian or vegan patients to meet nutritional requirements - Refer to dietitian and/or psychologist |
| Behavioral history | - No history of eating disorders or ARFID | - Borderline cases- based on judgment of the MDT | - Major mental illness - Eating disorders - ARFID | - Unnecessarily prolonged dietary restrictions - Development of abnormal restrictive behavior - Development of food anxiety - Negative impact on quality of life | - Stop dietary therapy in case of -severe psychopathology - MDT that includes dietitian and psychologist |

CD- Crohn Disease; IBD- Inflammatory Bowel Diseases; IBS- irritable bowel syndrome; CRP- C- reactive protein; ESR- Erythrocyte sedimentation rates; FCP-Fecal Calprotectin; PEN- Partial Enteral Nutrition; ARFID- Avoidant Restrictive Food Intake Disorder; MDT- Multidisciplinary team
